# Comparison of laparoscopic sleeve gastrectomy leak rates in five staple-line reinforcement options: a systematic review

**DOI:** 10.1007/s00464-019-06782-2

**Published:** 2019-04-16

**Authors:** Michel Gagner, Paul Kemmeter

**Affiliations:** 1 Department of Surgery, Hopital du Sacré Coeur, 315 Place D’Youville, Suite 191, Montréal, QC H2Y 0A4 Canada; 2 Herbert Wertheim School of Medicine, Florida International University, Miami, FL USA; 3 Westmount Square Surgical Center, Westmount, QC Canada; 4 Department of Surgery, Mercy Health Saint Mary’s, 2060 E Paris Ave SE #100, Grand Rapids, MI USA

**Keywords:** Bariatric, Metabolic, Laparoscopic sleeve gastrectomy, LSG, Leak, Staple line, Reinforcement, Systematic review

## Abstract

**Background:**

Staple-line leaks following laparoscopic sleeve gastrectomy (LSG) remain a concerning complication. Staple-line buttressing is largely adopted as an acceptable reinforcement but data regarding leaks have been equivocal. This study compared staple-line leaks in five reinforcement options during LSG: no reinforcement (NO-SLR), oversewing (suture), nonabsorbable bovine pericardial strips (BPS), tissue sealant or fibrin glue (Seal), or absorbable polymer membrane (APM).

**Methods:**

This systematic review study of articles published between 2012 and 2016 regarding LSG leak rates aligned with the Preferred Reporting Items for Systematic Reviews and Meta-Analyses guidelines. Variables of interest included leak rates, bleeding, and complications in addition to surgical and population parameters. An independent Fisher’s exact test was used to compare the number of patients with and without leaks for the different reinforcement options.

**Results:**

Of the 1633 articles identified, 148 met inclusion criteria and represented 40,653 patients. Differences in age (older in APM; *p* = 0.001), starting body mass index (lower in Suture; *p* = 0.008), and distance from pylorus (closer in BPS; *p* = 0.04) were observed between groups, but mean bougie size was equivalent. The overall leak rate of 1.5% (607 leaks) ranged from 0.7% for APM (significantly lower than all groups; *p* ≤ 0.007 for next lowest leak rate) to 2.7% (BPS).

**Conclusions:**

This systematic review of staple-line leaks following LSG demonstrated a significantly lower rate using APM staple-line reinforcement as compared to oversewing, use of sealants, BPS reinforcement, or no reinforcement. Variation in surgical technique may also contribute to leak rates.

**Electronic supplementary material:**

The online version of this article (10.1007/s00464-019-06782-2) contains supplementary material, which is available to authorized users.

Laparoscopic sleeve gastrectomy (LSG) has become the most commonly performed primary bariatric procedure performed in the United States (US) and worldwide [1, 2]. Since its early days of adoption, the complication of staple-line leak remains the greatest concern with the reported leak rates averaging 2.4% and ranging from 1.1 to 4.7% [3–8]. Over the past 10 years, multiple studies have attempted to identify parameters associated with decreasing the risk of leaks, which have included: varying bougie size, distance from the pylorus, surgeon experience, and reinforcement of the staple line [6, 9–12]. In regards to staple-line reinforcement, expert opinion from the International Sleeve Gastrectomy Consensus Conference in 2011 demonstrated that 77% of experienced LSG surgeons deemed staple-line buttressing as “acceptable” [7]. Multiple retrospective studies have further evaluated staple-line reinforcement, with the largest study published to date utilizing the Metabolic and Bariatric Surgery Accreditation Quality Improvement Program data base [10]. This study suggested that reinforcement of the staple line may actually be associated with increased leak rates, but the study results were limited by the lack of granular data to separate outcomes based on actual type of reinforcement utilized and the inclusion of discontinued material (i.e. glycolide diaxonone trimethylene carbonate, Duet TRS, Covidien, Norwalk, CT) [10].

In an attempt to provide buttressing-specific data, we previously reported the results of a systematic review of 88 articles published up to March 2012 with the purpose of comparing staple-line leak rates of 4 prevalent surgical staple-line reinforcement methods in 8279 LSG procedures. In that review, the overall leak rate was 2.1%, with the lowest rate in absorbable permeable membrane (APM) reinforced staple lines of 1.09% [13]. Our follow-up to this study included an additional 3416 APM-reinforced LSG patients and demonstrated that overall leak rates decreased to 0.67% from 2012 to 2015, perhaps suggesting a “learning curve” associated with the procedure [14].

Since the cutoff date for these previous reviews, the use of tissue sealants has become more prevalent. In this current systematic review, relevant articles of LSG and the use of staple-line reinforcement methods published from 2012 to 2016 are evaluated. The leak rates from 5 reinforcement methods of no reinforcement (NO-SLR), over sewing (Suture), bovine pericardium membrane (BPM), tissue sealant (Seal), and APM are evaluated.

## Methods and materials

### Search strategy, inclusion criteria, variables of interest

**Fig. 1:**
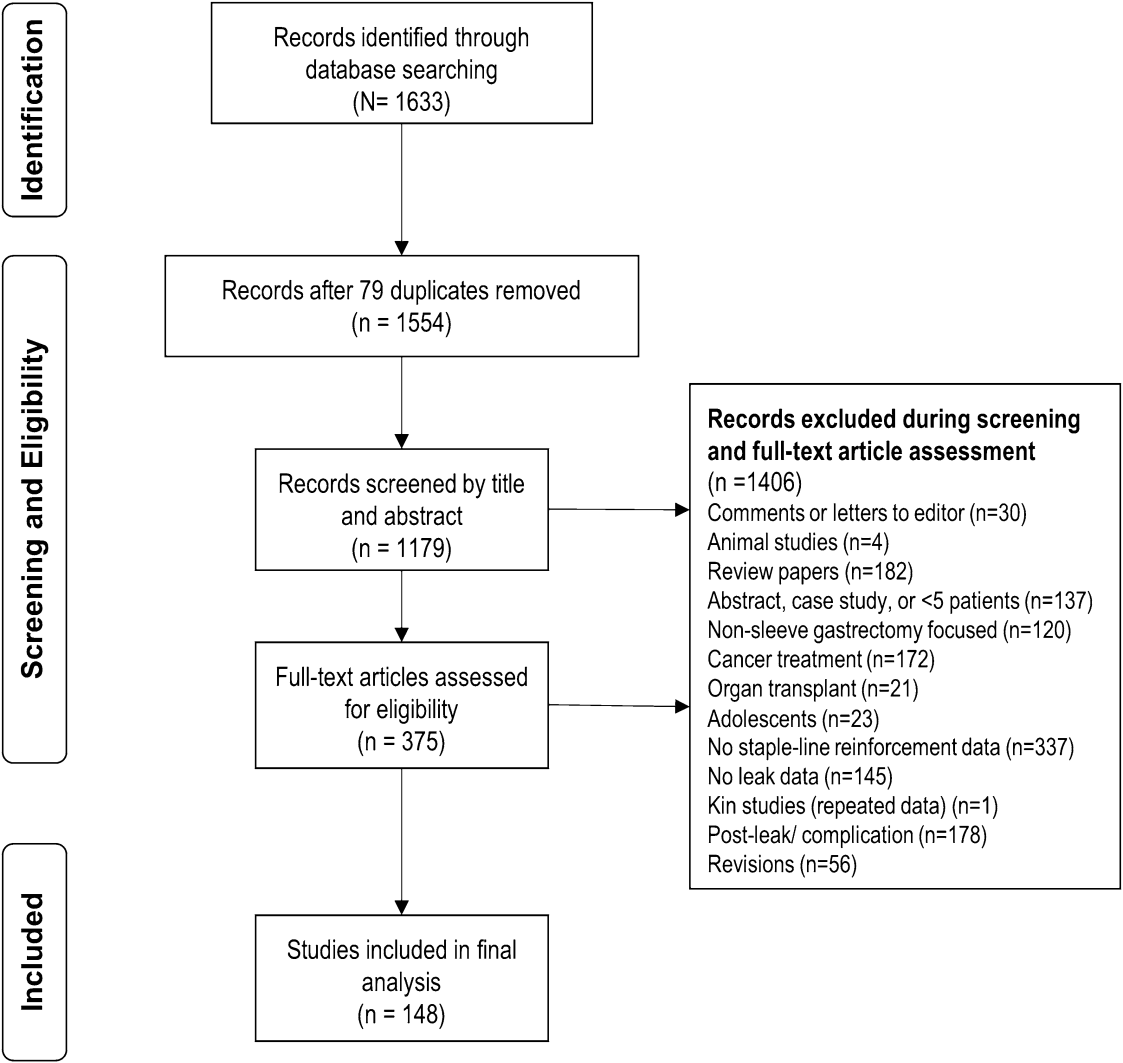
Search strategy

The search strategy used for this current review was consistent with our systematic review reported in 2014 and was aligned with the Preferred Reporting Items for Systematic Reviews and Meta-Analyses statement (PRISMA) [13, 15]. Briefly, the electronic literature search of the BIOSIS Previews^®^, Embase^®^, Embase^®^Alert, and MEDLINE^®^ databases with the keywords: “sleeve gastrectomy,” laparoscopic sleeve gastrectomy,” “vertical gastrectomy,” “leak,” “complication,” “morbidity,” or “fistula” limited to human patients and reports in English. The search period started from March 2012 through June 2016 (published or e-published ahead of print). Electronic results were screened by title to exclude duplicate studies and the remaining records were screened by reading abstracts. Full-text articles were included *only* if an LSG procedure, leak data, and type of staple-line reinforcement were reported. Of note, articles may have reported data for more than 1 reinforcement method of interest. As summarized in Fig. 1, excluded from eligibility were: Comments, Letters to the Editor, case reports series or studies with sample sizes of ≤ 5 patients, animal studies, review articles without accompanying data, and kin studies (i.e., reports with overlapping data or an author group that reported outcomes for similar periods of time). Analysis objectives centered on 5 reinforcement methods NO–SLR, suture, BPM, tissue sealant seal, APM and the number of patients with leak and without leak; bleeding, overall complications, and mortality were collected as text fields but not categorically summarized. Additionally, population and surgical variables of gender, age, body mass index (BMI), calibrating bougie size, and distance between the pylorus and gastric transection line were collected. Stapler types, staple heights, port type, number and placement, and other procedural characteristics were not included, as these details were not consistently reported.

### Abbreviated terms

No reinforcement = “NO-SLR”. Reinforcement by over sewing alone = “suture”. Reinforcement with nonabsorbable bovine pericardial strips (Peri-Strips Dry, Baxter^®^ Healthcare, St. Paul, MN) = “BPS”. Reinforcement with tissue sealant or fibrin glue (FloSeal or Tisseel fibrin sealant [Baxter^®^ Deerfield, IL, USA], Ifabond^®^ glue [Ifamedical, France], or Evicel^®^ glue [Ethicon™ Biosurgery, Inc., Somerville, NJ, USA] = “Seal”. Reinforcement with absorbable polymer membrane (GORE^®^ SEAMGUARD^®^, W. L. Gore & Associates, Elkton, MD, USA) = “APM”.

### Statistical analysis

Data were extracted by an individual from original sources to fields within an Excel (Microsoft, Redmond, WA, USA) database. Data manipulation and analysis was conducted using JMP statistical software, version 13.2.0 (SAS Institute Inc., Cary, NC). Criteria-based data were aggregated from selected studies representative of the 5 LSG reinforcement options of interest. Select demographic variables of age,  % females, and body mass index (BMI, kg/m^2^) and the surgical technique variables of bougie size and distance from pylorus were summarized using mean, standard deviation, range, and the percentage of studies reporting on each variable. The overall leak rate for LSG patients, as well as, patient leak rates within each of the 5 reinforcement categories were calculated. An independent Fisher’s exact tests was used to compare the number of patients with and without leaks for the different reinforcement options [16]. All statistical tests were 2-tailed and alpha was set at *p* < 0.05.

## Results

### Study characteristics

A total of 1633 articles were identified in the initial search. Figure 1 illustrates the identification, screening, and eligibility selection process. After removing duplicates (*n* = 79), the 1554 records were screened by title and abstract after which 1179 were excluded and the full-text articles for the remaining 375 records were assessed for eligibility. A total of 148 papers were included in the final analysis and the number of studies per reinforcement method were: 69 for NO-SLR  [9, 17–84], 70 for suture [9, 19, 20, 22, 26, 30, 46, 51, 53, 75, 76, 78, 83, 85–140], 9 for BPS [9, 72, 78, 83, 86, 89, 141–143], 9 for Seal [9, 30, 61, 74, 78, 103, 144–146], and 24 for APM [9, 39, 52, 53, 63, 89, 103, 131, 147–162]. Studies included in the analysis were comprised of 11 case series, 22 prospective randomized studies, 29 prospective studies, 1 randomized clinical trial, and 85 retrospective reviews and were conducted in Western Europe (*n* = 58), the US (*n* = 33), and other regions (*n* = 57; i.e. Asia and Middle East). Table 1 describes the study characteristics by reinforcement method and the associated article reference which reflects “double-counting” of an article in cases when more than 1 reinforcement method was reported for an article.

**Table 1:** Characteristics of accepted studies by reinforcement method groups

	Reinforcement method				
	NO-SLR	Suture	BPS	Seal	APM
Variables					
Publication date range	2012–2016	2012–2016	2012–2015	2012–2015	2012– 2016
Study design type^a^					
Case series	2	7	2	1	2
Prospective randomized	13	12	2	4	1
Prospective	11	17	1	2	5
Randomized clinical trial	0	1	0	0	0
Retrospective review	43	33	4	2	16
Total	N = 69	N = 70	N = 9	N = 9	N = 24
Region, n (%)^a^					
Other	26 (36.1)	38 (52.8)	4 (0.06)	3 (0.46)	1 (0.01)
United States	10 (27.0)	15 (40.5)	1 (0.03)	1 (0.03)	10 (27.0)
Western Europe	33 (45.8)	17 (23.6)	4 (0.06)	5 (0.07)	13 (18.1)

*APM* absorbable polymer membrane, *BPS* bovine pericardial strips, *NO*-*SLR* no staple-line reinforcement, *n* number of studies per reinforcement type, *N* number of studies overall, *NR* not reported, *P* prospective, *R* retrospective, *RCT* randomized controlled trial, *seal* tissue sealant, *suture* oversewing alone

^a^N = 148 for total number of citations included in analysis and N = 181 for total reinforcement outcome results and reflects some articles that were double counted for report of more than 1 reinforcement method

### Patient characteristics

The final analysis consisted of 40,653 patients from the 148 papers. At least one of the three patient characteristics variables (age, gender, or starting BMI) were reported in all but the following eight studies representing 12,473 patients [24, 28, 49, 63, 76, 97, 144]. In 8 additional studies, only one of the three patient characteristic variables were reported. Age was not reported for 40 patients in one study [43]; gender was missing for 1103 patients in 5 studies [42, 52, 143, 159, 161]; and starting BMI was not reported for 45 patients in two studies [25, 162]. Overall, patients had a mean age of 41 years, a mean starting BMI of 46.1 kg/m^2^, and 74% were female. Among studies reporting characteristics, differences were noted among the five reinforcement method groups in that patients in the APM group were older (45.6 ± 3.2 kg/m^2^; *p* = 0.001) and the starting BMI was lower for the suture group (43.7 ± 4.3 kg/m^2^; *p* = 0.008). The gender ratio in this analysis data set was similar across the reinforcement groups (Table 2).

**Table 2:** Characteristics of patients reported in accepted studies by reinforcement method

	Reinforcement method					*p* value
	NO-SLR	Suture	BPS	Seal	APM	
Variables mean ± SD (range^a^) [% reported^b^]						
Age, years	39.9 ± 5.2 (29.9–54.3) [90%]	41.1 ± 5.4 (27.0–64.1) [96%]	38.6 ± 5.5 (31.5–45.6) [100%]	39.8 ± 3.9 (32.3–44.1) [89%]	45.6 ± 3.2 (41.0–54.5) [92%]	0.0009
Female, %	75.9 ± 8.8 (47.0–100.0) [88%]	75.2 ± 8.6 (43.0–95.0) [96%]	73.2 ± 8.3 (40.0–86.0) [89%]	71.3 ± 14.9 (39.0–92.0) [89%]	73.2 ± 11.5 (10.0–100.0) [79%]	0.7608
Starting BMI, kg/m^2^	44.5 ± 4.9 (32.6–66.0) [90%]	43.7 ± 4.3 (34.9–68.4) [96%]	47.0 ± 3.4 (42.0–51.0) [100%]	47.9 ± 7.7 (42.1–65.0) [89%]	47.4 ± 3.3 (40.1–55.5) [88%]	0.0079

*APM* absorbable polymer membrane, *BPS* bovine pericardial strips, *N* number of patients, *NO*-*SLR* no staple-line reinforcement, *seal* tissue sealant, *suture* oversewing alone

^a^Minimum to maximum

^b^Percentage of studies that reported variables

### Surgical technique

The mean bougie size ranged from 36 Fr (NO-SLR and suture groups) to 34.6 Fr (Seal group); the differences between reinforcement type groups were not significantly different. The mean distance from pylorus ranged from 3.2 cm (BPS group) to 5.0 cm (suture group) and the difference was significantly different (*p* = 0.04) (Table 3).

**Table 3:** Bougie size and distance from pylorus by reinforcement method

	Reinforcement method					
	NO-SLR	Suture	BPS	Seal	APM	*p* value
Variables mean ± SD (range^a^) [% reported^b^]						
Bougie size (Fr)	36.1 ± 2.1 (30.0–50.0) [97%]	36.2 ± 7.2 (27.0–60.0) [93%]	35.1 ± 3.1 (32.0–40.0) [89%]	34.6 ± 4.7 (26.4–40.0) [78%]	35.7 ± 2.4 (29.0–42.0) [92%]	0.9834
Distance from pylorus (cm)	4.8 ± 1.1 (1.5–6.5) [90%]	5.0 ± 1.6 (1.5–10.5) [89%]	3.2 ± 0.4 (3.0–4.0) [67%]	3.9 ± 1.1 (3.0–5.5) [67%]	4.8 ± 0.8 (3.0–6.0) [79%]	0.0362

*APM* absorbable polymer membrane, *BPS* bovine pericardial strips, *max* maximum, *min* minimum, *N* number of studies reporting variables, *NO*-*SLR* no staple-line reinforcement, *seal* tissue sealant, *suture* oversewing alone

^a^Minimum to maximum

^b^Percentage of studies that reported variables

### Staple-line leak rate

A total of 607 leaks were reported in 40,653 patients yielding an overall leak rate of 1.49% (Table 4). The percentage of leaks was significantly lower for the APM reinforcement method (0.73%) compared with and in ranking order, suture (1.21%; *p* = 0.007), NO-SLR (1.89%; *p* < 0.0001), Seal (1.89%; *p* = 0.027), and BPS (2.73%; *p* < 0.0001) (Table 4). The leak rate for the tissue sealant reinforcement method was comparable to that of no staple-line reinforcement (*p* = 0.271). When looking at only studies conducted in the US, the APM reinforcement method continues to have the lowest leak rate (0.39%) among the reinforcement methods evaluated (Table 4).

**Table 4:** Leak rate by reinforcement method

	Reinforcement Type					
	NO-SLR	Suture	BPS	Seal	APM	TOTAL *N* = 40,653
Study overall						
Leaks, *n*	314	222	34	7	30	607
Patients without leaks, *n*	16,318	18,092	1210	356	4070	40,046
Leaks, %	1.9	1.2	2.7	1.9	0.7	1.5
*P* value compared to APM^a^	< 0.0001	0.007	< 0.0001	0.0271	–	–
United States only						
Leaks, *n*	14	23	4	1	9	
Patients without leaks, *n*	1059	3175	265	54	2302	
Leaks, %	1.30%	0.72%	1.49%	1.82%	0.39%	

*APM* absorbable polymer membrane, *BPS* bovine pericardial strips, *NO*-*SLR* no staple-line reinforcement, *seal* tissue sealant, *suture* oversewing alone

^a^Two-tailed Fisher’s exact test

## Discussion

Laparoscopic sleeve gastrostomy is a popular operation, and in the US, LSG has surpassed Roux-en-Y gastric bypass because of more favorable outcomes of lower mortality and overall morbidity, similar weight loss, and resolution of health comorbidities at 5 years [163–166]. Further supporting LSG as a preferred procedure is the lower leak rates, the twofold lower complication rate, and a mortality rate that is half that of Roux–en–Y gastric bypass [167]. Our current meta-analysis of 148 articles gathering data on 40,653 LSG patients, demonstrates an overall leak rate of 1.5% among the 5 staple-line reinforcement methods evaluated. Reinforcement with APM had the lowest statistically significant leak rate at 0.7% (*p* ≤ 0.007) despite a patient population that was older (*p* = 0.0009) and with a higher BMI [suture alone group had lower starting BMI (*p* = 0.0079)], both notorious as factors contributing to higher leak rates  [168].

The variability in staple-line leak rates among the five reinforcement types indicates that the type of reinforcement material is an important factor related to this complication. When comparing the leak rates from the current analysis to the previous review, it is interesting to note the reliability of the data between both studies [13]. Although leak rates have decreased among all reinforcement types, the overall propensity is the same: APM had the lowest (0.73% vs 1.09%) followed by suture (1.21% vs 2.04%), NO-SLR (1.89% vs 2.60%), and BPS (2.73% vs 3.30%)  [13]. Though tissue sealants were not evaluated in the previous review, it should be noted in the current study that Seal and NO-SLR methods had similar leak rates and that the addition of tissue sealants in the analysis did not alter the trend of lower leak rates. We speculate that the temporal reduction in leak complications in LSG is most likely related to surgical experience since there have been minimal-to-no-changes in the buttressing material from the previous to the current review. Two studies have demonstrated that surgeon technique and skill is associated with improved outcomes following bariatric surgery [11, 169]. Improvements in surgical techniques include: improved dissection with preservation of healthier and more vascular tissue by reducing thermal injury and tissue trauma, selection of appropriate staple height to accommodate tissue thickness, avoidance of narrowing near the angularis incisura, choice of adequate bougie sizes, and avoidance of stapling along the esophagus. If this is indeed the case, there is the possibility that further reduction in leak complications could be gained by improving intraoperative strategies. It has previously been reported by the Michigan Bariatric Surgery Collaborative that more experienced and higher volume surgeons use intracorporeal suturing more frequently [11]. This trend may have occurred in this current study and resulted in lower leak rates in the suture group. Indeed, a recent randomized study comparing the use of a running suture with invagination to no reinforcement demonstrated a reduction in leak rates for the suturing approach, although this came at a cost of higher operative time by 18 min [170]. Increased operative time and cost with intracorporeal suturing is supported in other studies that reported an additional 13 to 24 min per case. Additionally, there is evidence that staple-line buttressing with APM may actually be more cost effective at 6-months post-surgery [39, 171]. As APM and Suture were the two reinforcements with the lowest leak rates, this comparison warrants further study.

This current review highlights that the leak rate for studies conducted in the US were lower than the overall average leak rate of all studies evaluated. The APM was associated with the lowest leak rate when looking only at studies conducted in the US (0.39%) versus all studies in all geographic locations reported (0.73%). Indeed, every reinforcement method had a substantially lower leak rate in the US studies compared to the overall publications, with the exception for the Seal group (1.8% versus 1.9%, respectively).

The present study had many limitations. Inherently, the nature of the review method itself is a limitation as it relies solely on data provided within the publication. Further, this systematic review included only one randomized-controlled trial that met our review criteria. The collection of granular data such as the use of reinforcement on the entire staple-line versus selective areas, the use of buttressing material on both the cartridge and anvil side versus one side or the other, stapler type, and staple height would have been beneficial. Additionally, this study did not include a discontinued variety of 100% PGA APM or a recently available variety of 100% PGA APM due to a lack of sufficient publications.

This study was not designed to evaluate costs in relation to leak or bleeding complication. It is known that leaks are extremely costly, and for example, can result in prolonged hospitalization within an intensive care unit as well as additional outpatient costs [172]. Since bleeding complications can be associated with leaks, data regarding bleeding would have been an asset. Unfortunately, these data were inconsistently reported and thus were collected as free text which could not be categorically summarized. As mentioned previously, staple height selection was not uniformly collected, but might be a significant factor associated with staple-line leaks. Thick gastric tissue (i.e. antrum) is at risk of crush injury with too short a staple load, with incomplete staple formation which would fail to close the gastric resection margin, and thin gastric tissue (i.e. cardia) is at risk of loose staple-line formation with too tall staple load. With most leaks occurring on the proximal staple line near the gastroesophageal junction, it is possible that the thinner wall is at risk of injury related to uneven staple compression or inadequate compression to approximate the tissues. Other elements may be responsible, like ischemia and morphology. Buttressing material has been shown to more evenly distribute the staple pressure over a wider surface area thus resulting in higher burst pressures and lower bleed rates [173–179]. As such, we hypothesize that the lower leak rate associated with the use of a thin buttressing material, such as APM (0.5 mm maximum total thickness), is related to improved staple compression, given, of course, appropriate staple height selection. Conversely, we speculate that the variable thicker BPS reinforcement (0.4 mm - 1.2 mm) could result in variations of tissue compression, potentially resulting in a segment of staple line that is either too tight or too loose.

## Conclusion

Systematic review of 148 included studies representing 40,653 patients found that the leak rate in LSG was significantly lower using APM staple-line reinforcement than oversewing, BPS reinforcement, or no reinforcement. Selected operative strategies can result in lower leak rates after sleeve gastrectomy.

## Electronic supplementary material

Below is the link to the electronic supplementary material.

Supplementary material 1 (XLSX 40 kb)
